# Uniportal Thoracoscopic Debridement for Children With Refractory Pleural Empyema: Case Series of 21 Patients

**DOI:** 10.3389/fped.2021.777324

**Published:** 2021-11-24

**Authors:** Jin-Xi Huang, Qiang Chen, Song-Ming Hong, Jun-Jie Hong, Hua Cao

**Affiliations:** ^1^Department of Cardiothoracic Surgery, Fujian Branch of Shanghai Children's Medical Center, Fuzhou, China; ^2^Fujian Children's Hospital, Fuzhou, China; ^3^Fujian Maternity and Child Health Hospital, Affiliated Hospital of Fujian Medical University, Fuzhou, China; ^4^Fujian Key Laboratory of Women and Children's Critical Diseases Research, Fujian Maternity and Child Health Hospital, Fuzhou, China

**Keywords:** video-assisted thoracoscopic surgery, uniportal, children, pleural empyema, pyothorax

## Abstract

**Purpose:** The effectiveness of video-assisted thoracic surgery (VATS), even uniportal VATS (U-VATS), in the treatment of pleural empyema has recently been demonstrated. However, few works have evaluated its safety and feasibility for children. We review our experience with U-VATS in the treatment of pleural empyema for children under 11 years old.

**Methods:** From January 2019 to December 2020, we consecutively enrolled 21 children with stage II and stage III pleural empyema in our institution. A 1.0 cm utility port was created in the 5th intercostal space at the anterior axillary line. A rigid 30°5 mm optic thoracoscope was used for vision, and two or three instruments were used through the port. Surgery was based on three therapeutic columns: removal of pleural fluid, debridement, and decortication. A chest tube was inserted through the same skin incision. Perioperative data and outcomes were summarized.

**Results:** The procedures were successful, and satisfactory debridement of the pleural cavity was achieved in all cases. The mean age was 4.1 years (range: 6 months to 11 years old). The mean operating time was 65.7 ± 23.2 min. No intraoperative conversion or major complications were identified among the patients. The mean hospital stay was 5.0 ± 0.6 days. At a follow-up of more than 4 months after operating, all patients had recovered well without recurrence.

**Conclusion:** According to our experience, U-VATS debridement is feasible for the surgical management of stage II and III empyema in the pediatric population. Indeed, U-VATS permits easier performance and complete debridement and decortication, with a very low risk for conversion.

## Introduction

Pleural empyema is defined as the presence of purulent fluid in the pleural cavity. It is due to pleural space infection resulting from post-bacterial pneumonia in the majority of cases (1). According to its radiological (X-ray, computed tomography scan, and ultrasonography) features, empyema is classified into three stages (2): Stage I: Parapneumonic effusion, with an increase in pleural effusion; Stage II: Fibrinopurulent stage with loculations of pleural fluid and fibrinous septa formation; Stage III: Chronic organizing stage with scar adhesions and progressive constriction resulting in incarcerated lung. For patients with advanced disease (Stages II and III), early surgical intervention is beneficial to avoid more complex surgical procedures, higher morbidity, mortality, and longer disease duration (3). For pleural empyema in children: A systematic review of 44 retrospective studies comparing different treatment strategies (4). They were chest tube therapy (16 studies, 611 cases), chest tube with fibrinolytic drug (10 studies, 83 cases), video-assisted thoracic surgery (VATS) (22 studies, 449 cases) and thoracotomy (13 studies, 226 cases). This study found that patients who received VATS or thoracotomy had shorter hospital stays than those who were non-operatively treated. The American Association for Thoracic Surgery (AATS) recommends VATS debridement rather than open thoracotomy for the surgical management of empyema in the pediatric population (5).

Articles on surgical vs. non-surgical treatment have been widely reported in recent years. A 2017 meta-analysis (6) included eight randomized controlled trials with a total of 391 participants. The authors concluded that there was insufficient evidence to assess the impact of fibrinolytic therapy and that VATS may reduce the length of hospital stay compared to thoracostomy drainage. To date, very few works have evaluated the role of the uniportal VATS (U-VATS) approach in the treatment of pleural empyema in children, even though it currently represents the most innovative and less invasive thoracoscopic approach (7). We report our experience with U-VATS in the treatment of pleural empyema in children.

## Patients and Methods

The present study was approved by the ethics committee of our hospital and adhered to the tenets of the Declaration of Helsinki. Additionally, written informed consent was obtained from the parents of the patients.

### Patients

**Figure 1:**
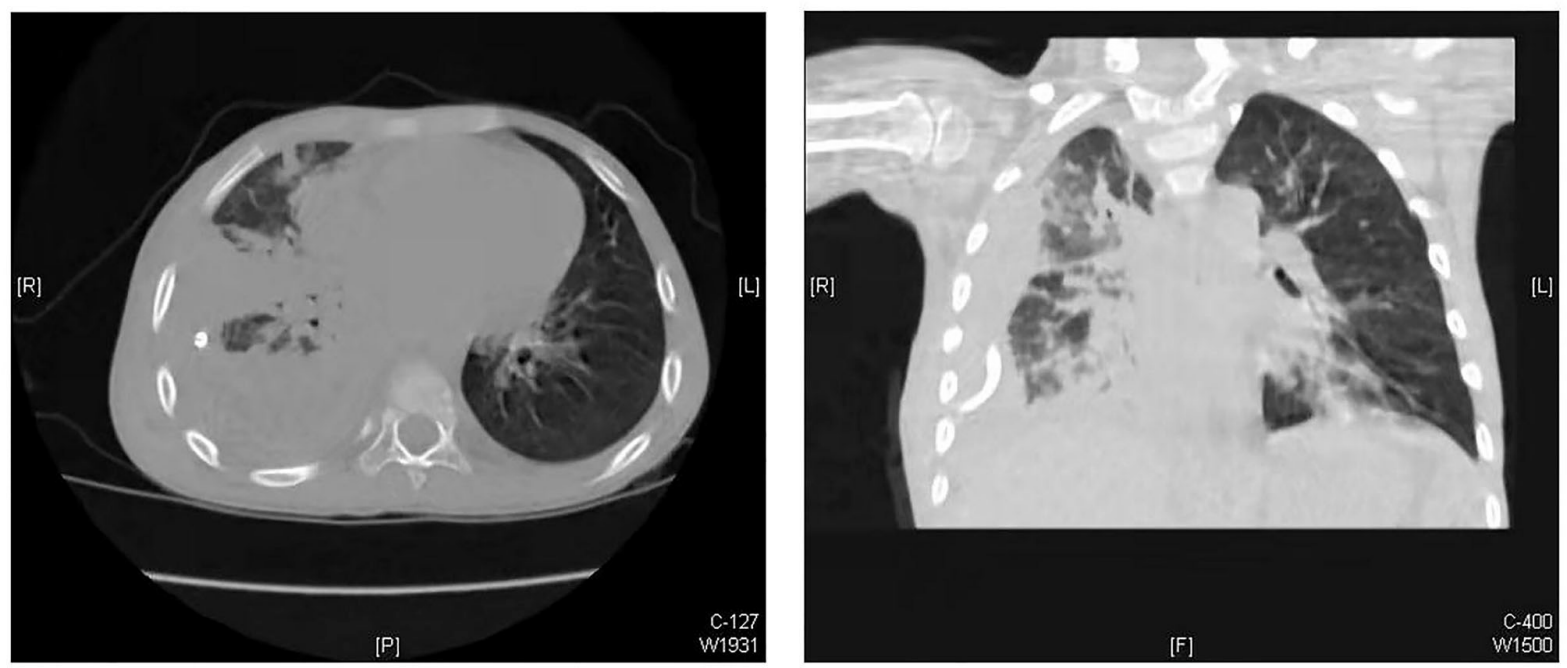
CT image of pleural empyema.

**Figure 2:**
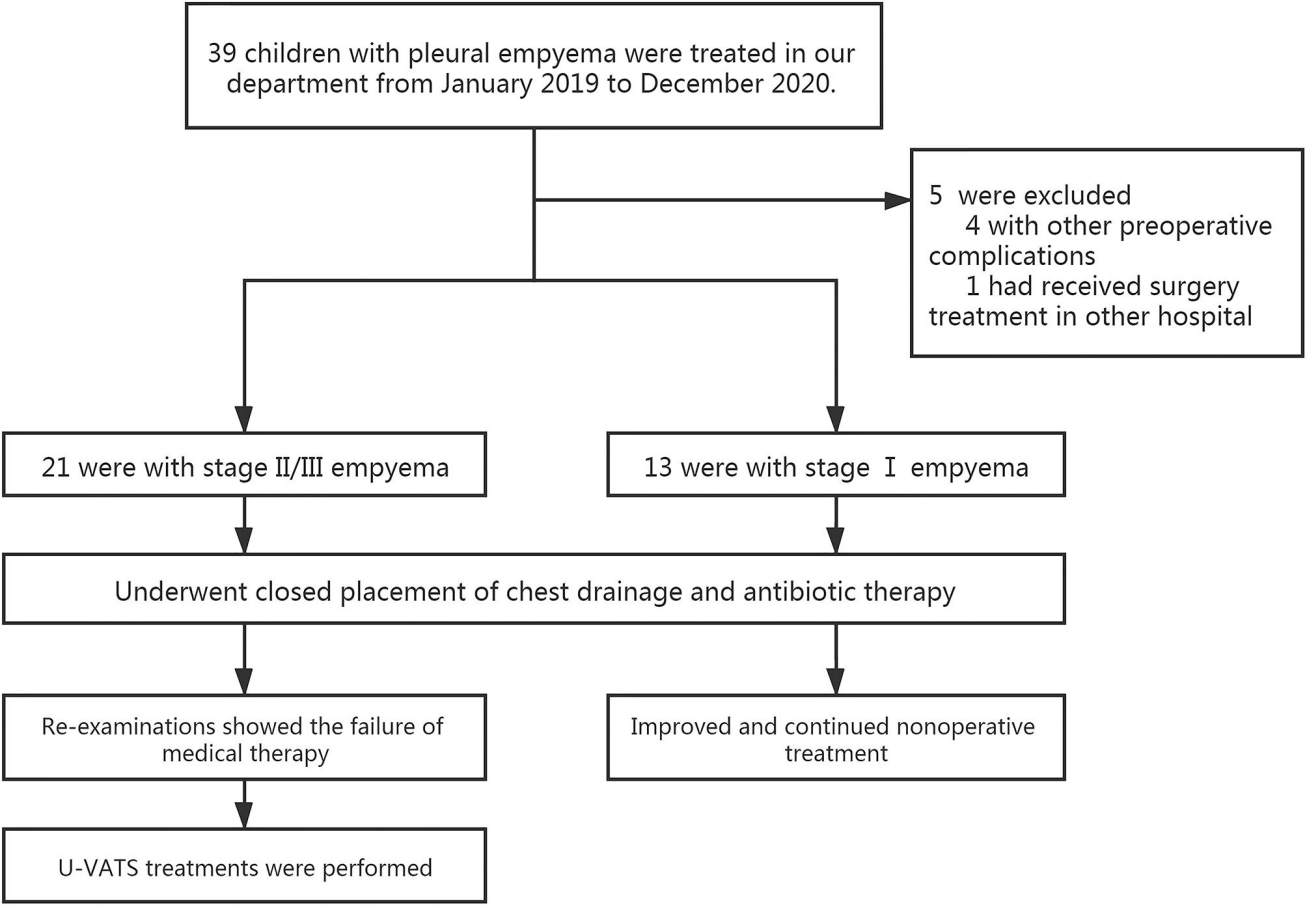
CONSORT flow diagram of participants.

From January 2019 to December 2020, 39 children with empyema were treated in our department. Our inclusion criteria were as follows: (1) patients with primary pleural empyema; (2) no surgical treatment received before admission to our hospital; and (3) Stage II and Stage III empyema with no improvement after conservative treatment. The exclusion criteria were patients with other pre-operative complications, such as congenital heart disease, immunocompromised state, restrictive or obstructive chest wall disease, and patients with additional foci of infection. The diagnosis and the stages of empyema were confirmed by chest X-ray plus ultrasound and then further verified by computed tomography (CT) scan (Figure 1). Thirteen of them had Stage I empyema, which improved after chest drainage and anti-infection treatment. One child was referred to our hospital after surgery in another hospital. Four children had other pre-operative complications. Once the diagnosis of pleural empyema was determined, a further ultrasound scan was performed to identify the presence of fibrinous septa and loculated fluid. Patients underwent closed placement of chest drainage and antibiotic therapy as the first procedure. If the following clinical and radiological examinations showed the failure of medical therapy with persistence of septic status, trapped lung or several loculations of pleural fluid and the presence of fibrinous septa, U-VATS treatments were performed. Finally, after eliminating contraindications such as the inability to tolerate one lung ventilation and severe coagulopathy (5), 21 patients received U-VATS during the study period (Figure 2). Their clinical information is summarized in Table 1.

**Table 1:** Demographical and clinical characteristics of the patients.

**Patient number**	**Age (years)**	**Gender**	**Weight (kg)**	**Location**	**Empyema stage**	**Duration of post-operative fever (days)**	**Operative time (min)**	**Drainage duration (days)**	**Hospital stay (days)**	**Post-operative complications**
1	3	F	15	R	Stage II	13	60	3	4	No
2	4	M	16.5	L	Stage II	15	60	4	5	No
3	11	F	24	L	Stages III	12	110	3	4	No
4	3	F	13.9	R	Stage II	13	120	4	5	No
5	10	M	22	R	Stages III	14	100	4	5	No
6	2	M	12.5	L	Stage II	15	50	3	4	No
7	2	M	13	R	Stages III	12	55	3	4	No
8	6	M	21	L	Stage II	13	60	5	6	Subcutaneous emphysema
9	3	M	15.5	L	Stages III	10	65	5	6	Pneumothorax
10	5	M	19	L	Stage II	14	70	4	5	No
11	1	M	9	R	Stages III	10	95	4	5	No
12	1	F	11	R	Stage II	14	75	5	5	No
13	0.6	M	7	R	Stages III	10	120	5	6	No
14	0.5	F	6.5	L	Stages III	12	100	4	5	No
15	3	M	14.5	L	Stages III	10	50	4	5	No
16	2	F	13	R	Stage II	12	55	5	5	No
17	5	F	20.5	R	Stages III	8	60	4	5	No
18	7	M	25	L	Stage II	12	65	5	6	Subcutaneous emphysema
19	6	M	22	L	Stages III	8	70	4	5	No
20	9	F	23	R	Stages III	13	100	3	5	No
21	3	F	11	R	Stages III	12	50	3	5	No
	4.1 ± 3.0	M: 57.1%	15.9 ± 5.5	R: 42.9%	Stage II: 42.9%	12 ± 2.0	65.7 ± 23.2	4 ± 0.76	5 ± 0.62	3 (14.3%)

### Surgical Technique

All patients were consecutively operated on by the same team of thoracic surgeons. We used a uniportal approach as described and defined by Migliore et al. (8). All patients were operated on under general anesthesia with selective one-lung ventilation using a single-lumen endotracheal tube with a bronchial blocker (Tappa, Hangzhou, China). The patients were positioned in the full lateral decubitus position, and the surgeon always stood at the ventral side of the patient. A 1.0 cm utility port was created in the 5th intercostal space (ICS) at the anterior axillary line. A wound protector (disposable wound protectors, Changzhou Anker Medical Co., LTD, Changzhou, China) was applied at the utility port. A rigid 30° 5 mm optic thoracoscope was used for vision, and two or three instruments, such as suction/irrigator devices, electrocantery, endoscopic graspers or scissors, endoscopic ultrasonic scalpels or open surgical instruments, were used through the utility port. The operation proceeded with septal rupture, debridement, and removal of all adhesions and inflammatory effusions from the diaphragmatic and parietal pleura to the apex of the chest cavity with the aim of creating a unique pleural cavity (without septa and loculations) and restoring the physiological movement of the lung. Multiple washings with warm physiological solution were carried out to eliminate all the residual effusion and organized pus from the visceral pleura. Under thoracoscopic control, lung inflation was performed to evaluate the efficacy of decortication. One chest tube was placed for pleural drainage after surgery. The chest tube was removed when there was no air leak, and when the amount of daily drainage was <1 mL/kg (9). Patients were discharged 1 day after removal of the chest tube if the follow-up chest X-ray showed no signs of pneumothorax and no signs of complications.

### Statistical Analysis

Continuous data are presented as the mean ± standard deviation and range, while categorical variables are presented as frequencies (%). SPSS Statistics (Windows version 19.0 IBM Corp., Armonk, NY) was used for analysis.

## Results

The mean age of the patients was 4.1 years (range 6 months to 11 years old), and 57.1% (12 patients) were males. Twelve patients (57.1%) presented Stage III empyema, and 9 patients (42.9%) presented Stage II empyema. All cases were related to complicated parapneumonic effusion. Pleural culture was positive in 6 (28.6%) patients. The main aetiologic agents were Staphylococcus aureus (2 patients), Streptococcus pneumoniae (3 patients), and Streptococcus viridans (1 patient). Of the 2 patients with Staphylococcus aureus, 1 had methicillin-resistant strain. All patients were treated with broad-spectrum antibiotic therapy. A chest tube was placed to evacuate pleural effusion, provided microbiologic insights. No fibrinolytic therapy had been administered. Among patients treated with chest tube insertion, 19 patients (90.5%) showed a trapped lung. All patients were treated with antibiotics plus thoracic drainage for at least 2 weeks (range 14–21 days), and with the failure of these medical treatments, the operation was carried out. The mean operation time for the U-VATS approach was 65.7 ± 23.3 min. Complete debridement and decortication were obtained in all patients, and no conversion or further access was needed for any reason. Post-operative complications occurred in 3 patients, including pneumothorax (air leakage >2 days) in 1 patient and subcutaneous emphysema in 2 patients. The drainage tube was removed after 4.0 ± 0.8 days, and patients were discharged after 4.9 ± 0.7 days. The long-term outcome was excellent in all cases, and all patients were alive with no recurrence.

## Discussion

To our knowledge, this is the first report of children with pleural empyema undergoing U-VATS for treatment. In 2001, Waller and his colleagues (10) concluded that VATS was proven to be as effective as open surgery in the treatment of Stage III empyema and had the advantages of being minimally invasive, plus reduced pain and hospitalization. Some authors (11–14) reported that the clinical effect of VATS decortications was better than that of thoracotomy. A recent meta-analysis (15) reviewed 14 published articles in 2010, and the results showed that VATS was superior to open surgery in terms of post-operative pain, complications, morbidity, 30-day mortality, and length of stay, with no significant difference in recurrence rates. U-VATS was first reported by Rocco in 2004 and has often been performed (16). Mahmoud Ismail and his colleagues (7) concluded that U-VATS allows for an easier performance, complete debridement and decortication, with a very low risk for conversion and excellent post-operative outcomes in terms of less pain, faster recovery, and cosmetic results. However, beyond that, few studies have evaluated the usefulness of the U-VATS method in the treatment of pleural empyema in children, and all the above reports are adult cases. Hung Do Manh and his colleagues (17) described a single trocar thoracoscopic surgery for pediatric pleural empyema, in which a 10 mm trocar was inserted with a scope and a single instrument for pleural dissection. Although different from the U-VATS as defined (8), some good results have been achieved. We tried to use a similar method and found that the main difficulty was that just one instrument could not perform debridement while aspirating the blood and pus at the same time.

**Figure 3:**
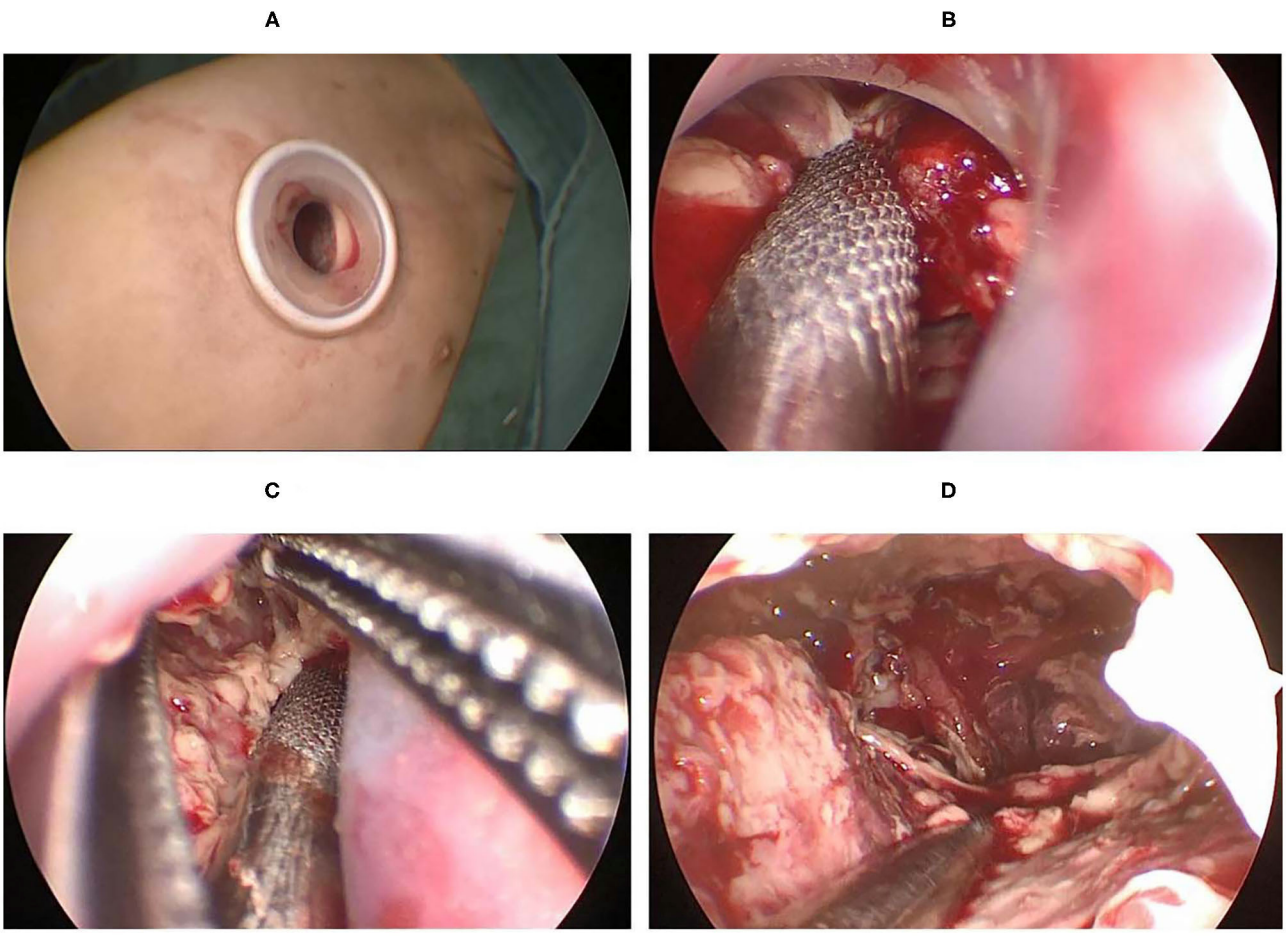
**(A)** A 1.0 cm port created in the 5th ICS at the anterior axillary line. **(B)** The aspirator was used to suck and remove the tissue around the incision. **(C)** Debride the area around the incision with aspirator and curved forceps. **(D)** Create a suitable surgical space.

Martin-Ucar and Socci (18) reported the advantages of U-VATS in many factors, including post-operative pain, post-operative recovery, view of the thoracic cavity, angle of vision, no rotational effect, and the ergonomic position of the operating surgeon. When performing U-VATS for children with pleural empyema, we noticed the above advantages and found some others from our single-center experience. How to expose the surgical field was the first question we faced. All the cases had severe pleural adhesions, and the surgeon needed to create a surgical space at the beginning of the operation. Tander et al. (19) reported a balloon-aided single-port thoracoscopic technique to achieve a wider field of vision, and a satisfactory conclusion was obtained. We found that the separation of adhesions from the edge of the incision using double-joint instruments of different curvatures could also gradually create a satisfactory surgical area (Figure 3).

There are some characteristics of the children's chest, including small cavum space and narrow intercostal space. The distance between the surgical area and the chest surface was short, which resulted in a limited angle of instrument activity. Angulated and narrow-shaft double-hinged instruments have become essential instruments for U-VATS, while articulating instruments help bring the operative fulcrum inside the chest. It is worth mentioning that, after early exploration, we found that a small incision is unique in its instrumentation requirement. Customized double-hinged surgical instruments with a 4-mm rod diameter were used in our U-VATS, and electrocantery was able to change the angle at will with a rod diameter of 2 mm. All the instruments made it advantageous to operate from multiple angles and to multiple regions.

Although pulmonary collapse would not be good due to pleural adhesion, we still recommend bronchial occlusion in our experience. Because the degree of lung collapse is gradually restored with debridement, without tracheal occlusion, the surgical space would be limited. There is a propensity for bleeding during empyema debridement and decortication, which can result in limited vision of the surgical field, making the procedure dangerous, especially near the hilum. We considered it safe and effective to perform debridement and aspirate the blood at the same time. When multiportal VATS or single-port VATS is performed, the chest is a closed environment. Although the surgical procedure is exercisable under artificial pneumothorax, the use of an aspirator would significantly reduce the surgical space, which is not a concern for U-VATS with tracheal occlusion. In addition, two or three instruments can simultaneously perform operative procedures such as suction, exposure, separation and hemostasis. Due to the limitation of retrospective studies, its superiority needs to be confirmed by further prospective research.

The application of the U-VATS technique marked a milestone innovation in thoracic surgery. However, there is often a long learning curve for the conversion from conventional multiportal VATS to U-VATS. Our surgeons have had many years of experience in U-VATS, so we conducted this study and aimed to evaluate the safety and feasibility of U-VATS for children with pleural empyema. As a result of the 21 successful cases mentioned, our experience was limited. We are also moving forward with more cases and more complex U-VATS procedures to further confirm our findings with prospective, comparative studies.

## Conclusions

The U-VATS approach is safe and feasible for children with pleura empyema. We presented U-VATS for the surgical management of Stage II and III empyema with satisfactory perioperative results.

## Data Availability Statement

The data of this study are available on request from the corresponding author. Requests to access these datasets should be directed to Hua Cao, caohua0791@163.com.

## Author Contributions

J-XH and HC made a significant contribution to the conception and design of the work. J-JH and S-MH drafted the work and revised it critically for important intellectual content. QC and J-XH are responsible for all aspects of the work and ensure that questions related to the accuracy or integrity of any part of the work are appropriately investigated and resolved. All authors read and approved the final manuscript.

## Conflict of Interest

The authors declare that the research was conducted in the absence of any commercial or financial relationships that could be construed as a potential conflict of interest.

## Publisher's Note

All claims expressed in this article are solely those of the authors and do not necessarily represent those of their affiliated organizations, or those of the publisher, the editors and the reviewers. Any product that may be evaluated in this article, or claim that may be made by its manufacturer, is not guaranteed or endorsed by the publisher.
